# Biodiversity Conservation, a Crucial Step Towards Food and Nutritional Security, Food Justice and Climate Change Resilience in Africa

**DOI:** 10.3390/plants14172649

**Published:** 2025-08-26

**Authors:** Olufunke Omowumi Fajinmi, Tafadzwanashe Mabhaudhi, Johannes Van Staden

**Affiliations:** 1 Research Centre for Plant Growth and Development, School of Life Sciences, University of KwaZulu-Natal Pietermaritzburg, Private Bag X01, Scottsville, Pietermaritzburg 3209, South Africa; rcpgd@ukzn.ac.za; 2 Centre for Transformative Agricultural and Food Systems, School of Agricultural, Earth and Environmental Sciences, University of KwaZulu-Natal Pietermaritzburg, Private Bag X01, Scottsville, Pietermaritzburg 3209, South Africa; mabhaudhi@ukzn.ac.za; 3 Centre on Climate Change and Planetary Health, London School of Hygiene and Tropical Medicine, London WC1E 7HT, UK

**Keywords:** biodiversity conservation, community inclusivity, ecosystem services, food security, climate change resilience

## Abstract

Biodiversity conservation has been identified as an important climate change mitigation tool. Healthy ecosystems act as natural carbon sinks while also strengthening resilience, making them essential for climate change adaptation. Climate change effects have led to various negative impacts, including biodiversity loss and food insecurity. The loss of forest biodiversity threatens vital wild fruits and vegetables that sustain rural communities, disrupting natural food sources and constituting a form of social injustice for poor, vulnerable, and previously marginalised groups in rural and semi-urban communities. Therefore, this study aimed to investigate the relationship between previous biodiversity conservation outcomes, ecosystem services, highly utilised wild vegetables and fruits, food and nutritional security, climate change effects, and climate resilience. We identified gaps in African biodiversity conservation and developed a conceptual framework to highlight integral principles required for the effective biodiversity conservation of wild forests in Africa. The integral principles are active community engagement, a strong network of stakeholders, sustainable plant resources management practices, legal reforms, and the creation of awareness through various platforms. Conservation policies should prioritise African indigenous wild, drought-tolerant vegetables and fruits that serve as an interface between food and medicine; play various roles in human survival in the form of ecosystem services; and act as carbon sinks to ensure a food-secure future with reduced climate change effects. The African indigenous community’s efforts in biodiversity conservation engagements are key to successful outcomes.

## 1. Introduction

Biodiversity refers to the variety of plants, animals, and microorganisms and their interactions. It is central to discussions at various levels due to its crucial role in supporting life on earth by providing essential resources and ecosystem services. Natures components such as soil, flora and fauna, and waterbodies exist synergistically to offer natural medicine, food, water, and oxygen, among other vital benefits, and biodiversity is the core of this intricate system. This interconnectivity highlights how the variety of life underpins the healthy functioning of the planet and human well-being. The COP26 summit held in Glasgow in 2021 underscored the critical importance of forest protection, culminating in a commitment to halt and reverse deforestation by 2030.

Biodiversity conservation laws have been identified as a crucial tool for protecting natural resources and ecosystems, thereby ensuring the sustainable use of indigenous bioresources to support human existence and survival. Deforestation, forest degradation, fragmentation, and climate change continue to threaten forest biodiversity, despite increased conservation efforts [1]. Country reports confirm that biodiversity loss, particularly through the degradation or loss of primary forests remains a persistent issue [1]. Globally, forest biomass carbon stocks have declined by approximately 11.1 Gt from 1990 to 2015, mainly due to forest conversion to agriculture and settlements, as well as land degradation; while global biomass stock declined by about 8 Gt from 1990 to 2020, with Africa experiencing the highest decline due to significant deforestation [2]. There is been a high rate of deforestation in Africa during the past half-century due to the changing structure of economies, the increasing population, and expanding globalisation [3]. Other major drivers of forest degradation in Africa are fuel wood collection and charcoal production [4], while pastoralism, small-scale farming, and the expansion of industrial tree crop estates have contributed greatly to deforestation [5]. Globally, the African continent has the second most significant deforestation in the tropics, with average per capita forest area declining from 0.8 ha to 0.6 ha per person from 1990 to 2015 [1]. The rate of net forest loss increased in each of the three decades after 1990 [6]. Between 2010 and 2020, Africa experienced the highest average (849,000 ha) annual loss of primary forest, an increase from previous decades [2]. Although deforestation rates have generally risen since 1990, a slight decline was noted between 2015 and 2020 [2].

In Africa, an increase of about 1 million ha was designated for the conservation of biodiversity per year from 2011 to 2015 [1]. Planted forest areas in Africa increased by 76,600 ha annually between 2010 and 2020, which was lower than the reported averages of 170,000 from 2000 to 2010 and 42,100 ha from 1990 to 2000 [2]. Plans for the management of forest exist for less than 25% of the forests in Africa [6]. Forest ecosystem services form integral parts of the support that the indigenous forests offer for the discovery of innovative therapeutic compounds, bioactive compounds for the development of functional foods, natural preservatives to enhance shelf life of agricultural products, overall supporting human survival from the cradle to the grave. The loss of valuable African indigenous medicinal plants has been identified as a major consequence of deforestation, prompting plant scientists to develop optimum propagation methods for the cultivation of these plants and thus reducing the pressure on the wild populations.

However, the negative impacts of deforestation have progressed into food and nutritional insecurity. Emerging evidence shows a link between deforestation, rising atmospheric CO_2_ levels, and declining agricultural productivity due to climate change [7]. Addressing deforestation in the region is therefore critical not only for mitigating climate change but also for ensuring long term food security and nutritional adequacy [8]. Globally, humanity is presently faced with an unusual biodiversity crisis that negatively impacts health and food security and the continuous ecosystem services forests offer to support human well-being [9]. In Africa, access to adequate and nutritious food remains a complex social challenge, influenced by poverty, rapid population growth, conflict, and inflation. Recently, various governments and donor agencies in sub-Saharan Africa have embarked on intervention programmes to improve food security [10]. Some of these interventions include the distribution of food materials and improving access to farm inputs [11,12], among others. Irrespective of these efforts, food insecurity is increasing in Africa [13] with many vulnerable groups still facing limited access to nutritious and balanced diets. These overwhelming issues of food and nutritional insecurity have been major barriers to the health and well-being of African rural populations [14].

### 1.1. Significance of the Study

In rural areas of Africa, a significant percentage of the rural population depends on indigenous leafy vegetables and fruits sourced from the wild as an alternative source of food and income for survival. Forests make significant, diverse and invaluable contributions to food security across Africa; however, increasing forest degradation and deforestation have led to the loss of several wild plants that serve as important interfaces between food and medicine. Hence, the deforestation of a reliable source of survival is an infringement of the rights of vulnerable groups. In Africa, approximately 7.2 million hectares of forest was lost annually between 2010 and 2020 [15]. This rapid rate of deforestation is a huge challenge to the continent’s forest ecosystems [16], increasing the loss of highly nutritious plant species with unique bioactive compounds, overwhelming poverty, higher levels of atmospheric carbon dioxide, and increased temperatures. These have been identified as global issues, exacerbated by climate change effects, which have been more severe in Africa, especially among low-income, poor and vulnerable groups. Hence, it is important to investigate the extent to which biodiversity conservation of indigenous forests and sustainability of forest ecosystem services support the survival of vulnerable poor communities in the face of climate change. The study aims to highlight the role of forests and forest ecosystem services in supporting human existence and survival, especially with the prevailing climate change effects and to develop a conceptual framework to highlight the integral principles needed to achieve effective biodiversity conservation in Africa. Therefore, the objectives of this study were to synthesise the literature in order to investigate

1. The methods of biodiversity conservation adopted in Africa and impacts on human survival;
2. Highly utilised African wild indigenous vegetables, climbers and trailers, and fruit trees and their roles in food and nutritional security, especially during drought and food scarcity;
3. The impact of community-inclusive biodiversity conservation methods on conservation outcomes and their various challenges;
4. The role of African indigenous wild fruit trees in carbon sequestration and climate resilience; and
5. The overall objective was to identify gaps in African biodiversity conservation and develop a conceptual framework to highlight the integral principles crucial for effective biodiversity conservation in Africa.

### 1.2. Methodology

A literature search was conducted using electronic databases such as Google Books, Google Scholar, Scopus, Wiley, Jstor, and the Web of Science. Other sources scrutinised for relevant information included published manuals, student theses from African universities and South African online news sources. These were supplemented by grey literature from government reports, policy documents, and international conservation organisations. Searched phrases included ‘ecosystem services of indigenous forests’; ‘old biodiversity conservation methods in Africa’; ‘recent biodiversity conservation methods employed in Africa biodiversity conservation’; ‘highly utilised African wild indigenous fruit trees and vegetables’; ‘drought-tolerant wild African leafy vegetables, climbers, trailers and fruit trees’, ‘wild edible climbers and trailers’; ‘wild African leafy vegetables and trailers that contribute to food and nutritional security in rural Africa’; ‘contribution of African communities in biodiversity conservation efforts and outcomes’; ‘outcomes of biodiversity conservation projects involving indigenous people’; ‘challenges involved in community based biodiversity conservation projects in Africa’; ‘challenges of community inclusivity in biodiversity conservation’; ‘African wild fruit trees that serve as carbon sequesters’; ‘carbon storage potentials of African wild fruit trees’; ‘role of indigenous forests in climate resilience’; and ‘drought-tolerant African wild leafy vegetables and fruits’.

This review did not follow a formal systematic framework such as PRISMA; however, a structured screening process was implemented to ensure transparency. The search was limited to literature published between 2000 and 2025 with the exception of publications with highly relevant information that has not been duplicated in any other documents. For the study selection, all retrieved records were screened in three stages: title screening to remove irrelevant studies, abstract screening to determine preliminary relevance based on scope and reported outcomes, and full-text review to confirm the inclusion of studies that met all criteria. Records were included based on their relevance to the aim and objectives of the study. Inclusion criteria included studies focusing on biodiversity conservation methods within Africa; studies reporting ecological indicators of success, such as habitat restoration outcomes; studies reporting highly utilised wild African indigenous leafy vegetables and fruits; and studies reporting mineral, nutritional and bioactive contents of highly utilised wild, African indigenous vegetables and their biological activities. In addition, studies reporting wild African indigenous fruits and vegetables consumed as food during drought and food scarcity; research work reporting drought-tolerant wild fruits and vegetables and their modes of survival; African indigenous trees that sequesters carbon; and climate change mitigation and resilience in Africa. At least one community-inclusive biodiversity conservation project per region in Africa (North, East, South and West Africa) was highlighted. Exclusion criteria included: opinion papers or purely theoretical discussions; publications lacking experimental accuracy, research integrity, empirical evidence or measurable outcomes; studies outside the African context; agronomic articles without reference to food and nutritional security, climate resilience and climate adaptation. A summary of the screening process is provided in Figure S1, while the inclusion and exclusion criteria are detailed in Supplementary Table S1. Approximately 260 peer-reviewed publications and grey literature sources were chosen for review. The information sourced from the various databases was used to identify gaps in biodiversity conservation in Africa and a conceptual framework was developed. 

## 2. Results and Discussions

### 2.1. Ecosystem Services

The four categories of ecosystem services according to Millennium Ecosystem Assessment [17] are as follows: provisioning services (food, fibre, genetic resources, biochemicals, natural medicines, ornamental resources, freshwater); regulating services (air quality regulation, climate regulation, water regulation, erosion regulation, disease regulation, pest regulation, pollination); cultural services (cultural diversity, spiritual and religious values, recreation and ecotourism, aesthetic values, knowledge systems, educational values); and supporting services (soil formation, photosynthesis, primary production, nutrient cycling, water cycling) [17]. This model of ecosystem services provides a framework that allows the use of biodiversity and other bioresources to be strategically optimised [18] to support the human population surrounding it. However, the strategic exploitation of bioresources to support human existence and survival can only be ensured by developing effective biodiversity conservation policies.

### 2.2. Biodiversity Conservation Policies/Methods and Outcomes

Contemporary conservation policies often involve the establishment of national parks and protected areas that deprive and exclude indigenous people and local communities from their ancestral lands [19]. This method of biodiversity conservation was established in the United States in the early 19th century and has denied indigenous peoples their rights [20], evicted them from their ancestral lands and triggered prolonged social conflict, starvation and death [21]. Hence, this mode of conservation is often referred to as “fortress” or “colonial” conservation [21,22]. This method is based on the opinion that biodiversity protection could only be achieved by creating protected areas where ecosystems can function in isolation from human disruption [23,24], assuming indigenous people and communities utilise natural resources unreasonably, causing damages resulting in biodiversity loss and ultimately, environmental degradation [24].

However, this method of conservation often deprives indigenous people of several ecosystem services that have previously supported their survival. This method prevents local people dependent on the natural resource base from accessing the resources, involves park rangers patrolling the boundaries, often using coercion and violence to enforce compliance with the policy; and only tourism, hunting, and scientific research are allowed within the areas [24]. The creation of national parks and game reserves does not fully allow for the opportunity to make use of forest biodiversity for the vulnerable and marginalised groups, as their major benefits from ecosystem services are food and medicine, which are an integral part of human existence and survival. According to Piccolo et al. [25], ecological justice and ecological values should be prioritised to ensure that the human population survive on earth. However, ecological values can be detrimental to social values [26] if conservation is prioritised over basic human rights [19]. According to Montgomery et al. [27], ‘social justice is integral to effective conservation’. Therefore, effective biodiversity conservation must align with human rights principles to avoid infringement on the human rights of communities; ensure social and economic equalities through access to forest resources and ecosystem services. Forest biodiversity provides irreplaceable sources of food and medicine [28], suggesting that the deforestation of indigenous forests, which contain plant resources used as valuable traditional medicines and food sources, is a social ill that affects the availability of free, wild foods and traditional medicines that support food and nutritional security and health.

### 2.3. Wild Indigenous Fruit and Vegetable Species with Provisioning Services (Food and Natural Medicine)

Limited access to nutritious food is a challenge among low-income groups, with inflation worsening the situation. In rural communities, foraging for wild plants has long served as a survival strategy. Africa’s forests are home to various wild leafy vegetables, climbers and trailers, fruit trees and nuts. Several of these plants are also utilised for medicinal purposes. There are certain wild plants used as food across Africa, while some only occur in certain parts of Africa. For example, Marula and some other African indigenous fruit trees occur across several African countries. Highly utilised African wild, indigenous fruit trees, vegetables, climbers and trailers mentioned in the literature are *Sclerocarya birrea* subsp. caffra (Sond.) Kokwaro, *Dovyalis afra* (Hook.f. & Harv.) Warb. (often referred to as *Dovyalis caffra* (Hook.f. & Harv.) Hook.f.), *Parinari curatellifolia* Planch. ex Benth., *Strychnos madagascariensis* Poir., *Strychnos spinosa* Lam.; Amaranthus (various species), *Corchorus olitorius* L., and *Cleome gynandra* L. (also referred to as *Gynandropsis gynandra* (L.) Briq.); and *Momordica balsamina* L., *Momordica charantia* L., *Citrullus colocynthis* (L.) Schrad. Plant names have been checked with Medicinal Plants Names Services “http://mpns.kew.org (accessed on 28 July 2025)” [29].

#### 2.3.1. African Wild Indigenous Fruit Trees with Various Ecosystem Services

*Sclerocarya birrea* (Marula) is a widely distributed [30], well-known indigenous plant in southern Africa and 25 other African countries [31,32]. Marula has become an important commercial commodity in the local and global trading arenas [33], thus increasing its demand. The single-stemmed Marula tree exists in 29 countries, with female trees bearing approximately 500 kg of fruit each year, while the male tree produces a magnificent floral display [34]. This is an indication that female trees are a source of food, while the male plant supports ecosystem services such as pollination. Marula fruit and nuts are used as food (Table 1), and they have various essential nutrients and minerals (Table 2). The utilisation of Marula fruit for food purposes could help obtain vital nutrients and overcome typical deficiencies in protein, amino acids, vitamins, minerals, and other essential nutrients that are often common in many rural diets [35].

Fermented and unclarified Marula fruit juice has been reported to contain reasonable amounts of amino acids and protein, which could increase protein intake [36]. Amino acids play a crucial role in health as they are the building blocks of proteins, protein complexes, and various important metabolites such as neurotransmitters [37]. Hence, consumption of Marula could support good health and vitality. For more than 10,000 years, the Marula fruit has been integral to diet, socioeconomic life and cultural traditions of communities across southern Africa [38]. *Sclerocarya birrea* and *Dovyalis caffra* are among the most preferred indigenous fruit trees in Northern KwaZulu-Natal [39]. Most southern African communities gather and consume the larvae of the Mopane worm (*Imbrasia belina*), a highly sought-after traditional food that hatches on the Marula tree leaves [40]. Almost all parts of the Marula tree are valuable and utilised, while their uses vary according to locations and tribes [41]. Marula tree potentials make it a valuable indigenous tree crucial for the sustainability of livelihoods. *Sclerocarya birrea* in African forests offers provisioning services (food and natural medicine) and cultural services (spiritual and religious values), among others (Table 1), and is more utilised than other wild fruits such as *Dovyalis caffra*.

**Table 1:** Provisional services of some selected wild African indigenous leafy vegetables and fruits.

Plant Specie	Provisional Services (Food and Medicinal Uses)
*Sclerocarya birrea* subsp. caffra (Sond.) Kokwaro	Fruit pulp and edible nuts are consumed [34] and used in the production of beverages and cosmetics [42]. Fruits are used to produce sweets, wine and flavourings [34]. Oil is extracted from the kernels for domestic use [43]. The oil is used to preserve meat and meat products [44]. Wood is used as construction material and leaves serve as fodder [45]. Root is used to treat an internal ailment (kati), while the bark is used to treat stomach disorders [46]. The bark decoction is used to treat dysentery, diarrhoea, and rheumatism and has a prophylactic effect against malaria [47]; and bark is used to treat haemorrhoids; roots and bark are used as laxatives, and a drink made from the leaves is used to treat gonorrhoea [47].
*Dovyalis afra* (Hook.f. & Harv.) Warb. (often referred to as *Dovyalis caffra* (Hook.f. & Harv.) Hook.f.)	The fruit is eaten raw by people and wild animals, or cooked, utilised as jelly, pickles and jam [48] and often used to produce juices and wine [49]. Its juice is added to boiled millet or sorghum porridge among the Bapedi people of South Africa [50]. The boiled fruit is used to flavour meat and fish and a brief simmering of fruits is performed, with products added to fillings used in pies, puddings and cakes [51].
*Parinari curatellifolia* Planch. ex Benth.	The plant is used for the treatment of cancer, pneumonia, fever, malaria, typhoid, hypertension, microbial infections, pain, anti-inflammation, and toothache [52,53]. The tree is a valuable source of food and ethnomedicines across Africa as a result of its rich nutritional content and phytochemicals in various parts of the plant [54]. The fruit is utilised as food and in the production of traditional alcoholic drinks [55,56].
*Strychnos madagascariensis* Poir.	Fruit pulp is consumed raw as a snack and processed into value-added food products (such as fruit rolls, powders, jams, or juices) [57,58].
*Strychnos spinosa* Lam.	Fruit is used to produce alcohol, fruit juice, and jam [59]. Fermented combinations of maize meal and *S. spinosa* pulp [59] and sorghum porridge mixed with *S. spinosa* pulp [60] are consumed in rural KwaZulu-Natal and Zimbabwe, respectively. Plant parts are used to treat snakebites, ulcers, wounds, headaches, gastric and intestinal problems, venereal diseases, leprosy, diarrhoea, and fever [61]. Leaf decoction combined with bark powder is used to treat wounds, while the dried powdered leaves are added to food to treat liver damage [61]. The green fruits are used as an antidote for snakebite [62,63]. Bark decoction is used to treat stomachache [59,64].
*Amaranthus* (various species)	Several studies have shown that oil extracted from *Amaranthus* seed or leaves can benefit those with cardiovascular disease and hypertension [65]. Vegetable *Amaranthus* has been a good source of medicine for young children, lactating mothers, and other patients with constipation and in addition, it is used to treat fever, anaemia, or kidney complaints [66]. Amaranth may present a potential source of cancer treatment as the seeds are a natural source of squalene [67], a valuable antioxidant known for anticancer activity [68]. Amaranth oil contains 6 to 8% squalene [69].
*Corchorus olitorus* L.	It is used to treat heart failure, diarrhoea, typhoid fever and colic [70]. Leaves are used to cure gonorrhoea, chronic cystitis, pain, fever, and tumours [71].
*Cleome gynandra* L. (also referred to as *Gynandropsis gynandra* (L.) Briq.)	Spider plant is used as a traditional medicine all over Uganda to hasten childbirth as a result of its uterotonic activity [72]. The entire plant has been utilised traditionally to treat a variety of diseases and conditions such as anaemia, arthritis, diabetes, cancer, piles, rheumatism, scurvy, tumours, cardiovascular diseases, chest pains, constipation, malaria, a relieving eyewash [73,74] migraine headaches, epilepsy [75] and stomach ache [76]. The extract is used to treat snake bites, food poisoning and severe pain caused by scorpion stings [77]. The sap of the leaves is used to manage severe threadworm infections and relieve cerebral pain [78]. Also, the sap from pounded young leaves is squeezed into the ears, nose, and eyes to control epileptic seizures and relieve earache [78]. The decoction of leaves and roots relieves fever and headaches and alleviates sexual weakness [79].
*Momordica balsamina* L.	Fresh leaves are consumed as vegetables [80]. Leaves are used to treat diabetes, jaundice, fever, gonorrhoea, tuberculosis, and viral infections [81].
*Momordica charantia* L.	The young fruits and shoots are consumed in some parts of West Africa, and they are used as an emmenagogue to facilitate childbirth in Ivory Coast [82]. It is used to treat diabetes, measles and chicken pox [83], tumours, wound, rheumatism, malaria, vaginal discharge and to expel intestinal gas, while the seeds are used to induce abortion [84,85]. In Nigeria and Ghana, the root of the plant is used as an abortifacient together with the fruit as well as an ingredient in aphrodisiac preparation [84].
*Citrullus colocynthis* (L.) Schrad.	In Northeastern Morocco it is used to treat various cardiovascular system diseases [86]. In East Africa, seed tar is applied to the skin by nomads. But, the digestion of this fruit results in acute toxic colitis, bloody diarrhoea, and changes in the colon [87]. In southern Tunisia, *C. colocynthis* is a useful medicine for gout, arthritis, and inflammatory disorders and its kernels are used in food preparation in many African countries [88].

**Table 2:** Nutritional, mineral and bioactive contents of some selected wild African indigenous leafy vegetables and fruits.

Wild Forest Species	Nutritional, Mineral and Bioactive Contents
*Sclerocarya birrea*	Marula pulp and nuts are rich in various healthy saturated fatty acids, such as tetradecanoic and hexadecanoic acid, and unsaturated fatty acids, such as oleic acid, linoleic acid, α-linolenic acid, and eicosanoid acid with cardioprotective activity [40,89,90].
*Dovyalis caffra* (Hook.f. & Harv) Hook.f.	The fruit is a valuable source of ascorbates [35,91]. Chlorogenic acid, catechin, and gallic acid are the main constituents of the fruit while hesperidin, rutin, ellagic acid, quercetin, kaempferol and apigenin were detected in lower quantities [92]. Fruits contain p-coumaric acid, p-hydroxyphenylacetic acid, 3-methoxy-4-hydroxyphenylacetic acid, m-hydroxybenzoic acid, vanillic acid [93], chlorogenic acid, procatechic acid [94], Pyrogallol and Catechin [95]. Kei–apple fruit juice also contains a high concentration of ascorbic acid [93].
*Parinari curatellifolia* Planch. ex Benth.	The fruit is rich in vitamin C, protein and calcium [55,56]. The leaves contain compounds such as alkaloids, flavonoids, and saponins [52], while the stem contains saponins, alkaloids, tannins, cardiac glycosides, flavonoids, digitalis glycosides, phenols, terpenes, and steroids [96].
*Strychnos madagascariensis* Poir.	Fruits have high sugar, fibre, potassium and iron content [97,98].
*Strychnos spinosa* Lam.	Fruit pulp contains fibre, carbohydrates, and vitamin C [99].
*Amaranthus species*	Amaranth seeds contain approximately 7% squalene [69]. Amino acid in Amaranth grain includes arginine, histidine, isoleucine, leucine, lysine, methionine, phenylalanine, threonine, tryptophan, and valine [100]. Phytonutrients in Amaranth grain include flavonoids, quercetin, nicotiflorin, rutin, ferulic acid, gallic acid, caffeic acid, p-coumaric acid, isoquercitrin, anthocyanins, syringic acid and vanillic acid [101]. Leaves contain high protein [102].
*Corchorus olitorus* L.	The leaves are rich in beta-carotene, iron, calcium, fibre, vitamins C, A, and E, proteins, sodium and folic acid [103], amino acids, and essential minerals [104]. The plant parts, such as the roots, bark, leaves and seeds, contain flavonoids, cardiac glycosides, fatty acids, triterpenoids, polysaccharides and phenolics [105,106,107,108]. Chlorogenic acid is present in leaves [109]. Other phenolic compounds present are 3,5-dicaffeoylquinic acid, quinic acid, gallic acid, protocatechuic acid, 4-O-caffeoylquinic acid, caffeic acid, 1,3-di-O-caffeoylquinic acid, feruloyl-quinic acids, and 4,5-di-O-caffeoylquinic acid, which were identified from the leaves of *C. olitorius* [110]. Trans-ferulic acid, p-coumaric acid, and rosmarinic acid were detected from *C. olitorius* [107,111]. The leaves contain flavones (cirsilineol and cirsiliol), flavones glycosides (apigenin, apegenin-7-O-glucoside), flavanones (naringenin, naringin), astragalin (kaempferol-3-O-β-D-glucopyranoside), tolifolin (kaempferol-3-O-β-D-galactopyranoside), and jugulanin (kaempferol-3-O-β-L-arabinopyranoside) [111].
*Cleome gynandra* L.	It has high α-carotene, α-tocopherol, β-tocopherol and γ-tocopherol, ascorbic acid, β-carotene, lutein, violaxanthin, and β-cryptoxanthin content [74]. Leaves contain magnesium, calcium, iron, and zinc [112]. It is rich in flavonoids and phenolics [113].
*Momordica balsamina* L.	The leaves, fruits, seeds, and bark of the *M. balsamina* contain alkaloids, flavonoids, glycosides, steroids, terpenes, cardiac glycoside, saponins, tannins and lectins [114]. Balsamin found in leaves, fruit, stem of *M. balsamina* has anticancer activity [115].
*Momordica charantia* L.	The leaves and flowers of *M. charantia* contain triterpenoids (momordicine and charantin), carotenoids (antheraxanthin, lutein, violaxanthin, α-carotene, and β-carotene), and phenylpropanoids (caffeic acid, chlorogenic acid, epicatechin, gallic acid, p-coumaric acid, rutin, and trans-cinnamic acid) [116]. The leaves of contain Momordicin I, Momordicin IV, aglycone of Momordicoside, aglycone of Momordicoside L and Karavilagenin D [117].
*Citrullus colocynthis* (L.) Schrad.	The major minerals in the seeds are calcium, magnesium and potassium [118]. Twelve alkaloids, including quinoline, nicotinamide, uracil, 2-hydroxyquinoline, 2-methylquinoline, 4-hydroxyquinoline, 4-methylquinoline, 6-hydroxyquinoline, 6-methylquinoline, 7, 8-benzoquinoline, 8-hydroxyquinoline, and 8-methylquinoline, were detected in *C. colocynthis* fruits [119]. *Citrullus colocynthis* contain ketones, epoxy compounds, hydrocarbons [120], and fatty acids [121].

Kei apple (*Dovyalis caffra*) is a beautiful evergreen fruit tree with aromatic apricot-like fruit, indigenous to the Kei River area [48]. *Dovyalis caffra* is an indigenous South African plant with a very nutritious, characteristic deep yellow colour and a sour-tasting fruit [49]. The tree is abundant in the wild in the Eastern Cape, KwaZulu-Natal, Mpumalanga and Limpopo provinces [122] and other southern African countries. The Agricultural Research Council—Institute for Tropical and Subtropical Crops have reported a decline in *D. caffra* among natural populations [48].

*Parinari curatellifolia* (Mobola plum) is an important evergreen tree indigenous to the Miombo woodland of south-central and eastern Africa [123]. The primary distribution in African countries includes West Africa, East Africa, and southern Africa [124]. The tree bears an orange-yellow colour fruit when fully ripe with a light yellow to reddish pulp, sweet taste and edible seeds [54].

*Strychnos madagascariensis* (black monkey orange) is a native southern African fruit-bearing tree, highly valued in rural communities [125]. This plant is valued for its high fruit yields, fruiting seasons, drought tolerance and wide distribution across the northern and eastern regions of southern Africa [97]. The black monkey orange fruit is well utilised in rural households, especially during food scarcity [97]. According to Van Rayne et al. [99], fruit trees such as *Strychnos* species are potential tools for alleviating food and nutrition insecurity and assisting in creating food products for consumption. *Strychnos innocua* Delile and *Strychnos spinosa* Lam. are species of the Loganiaceae family. *Strychnos innocua* is distributed from West Africa and East Africa, extending southward to Angola, Zambia, Zimbabwe and Mozambique [126]. Various parts of *Strychnos spinosa* is utilised for medicinal purposes [127]. It is among the most valued wild edible fruit trees [128] and an essential food resource utilised by poor farmers during periods of food scarcity [129,130] in Africa. It is the most important indigenous fruit tree species in KwaZulu-Natal, South Africa [39]. Irrespective of the enormous contribution of its fruits to the livelihood of many people in Benin, the gradual change in forest land use could lead to the population decline of both *S. innocua* and *S. spinosa* [131]. Among the 13 species of *Strychnos* reported in Benin [132], *S. innocua* and *S. spinosa* are commonly known and mentioned in the literature for their uses as food and roles in treatment of human and veterinary diseases [64,133,134]. *Strychnos* species have great socio-economic importance in Benin [135]. As a result of the great potential of the trees, farmers often keep some trees in their fields during land clearing [131]. Foot paths from indigenous forests and farms also offer a range of wild edible vegetables.

#### 2.3.2. Wild Edible Vegetables in African Forests

Wild-sourced edible vegetables serve as a source of food in rural communities, especially in developing countries. Rural households engage in foraging wild edible vegetables as a survival strategy [44]. Wild plants support dietary quality and meet critical nutritional needs, particularly in underserved areas. As natural food reserves, forests can provide a stable food supply during environmental and economic stress.

*Amaranthus* species are one of the most utilised wild vegetables in Africa [66,136,137,138,139,140,141,142,143,144,145,146,147,148]. Its ability to reseed and grow make it a potential regenerative plant for forest environment and a sustainable food resource. According to Emire et al. [149], *Amaranthus* species can survive unfavourable conditions such as inadequate moisture, and high temperatures. *Amaranthaceae* is among the families with the highest number of species recorded in the Eastern Cape Province [150]. Regular consumption of Amaranth seeds could prevent constipation due to their fibre content [151]. *Amaranthus* play a significant role in the food and nutrition security of vulnerable groups in rural communities [65]. Cultivated *Amaranthus was* discovered to have less protein and amino acids compared to the wild species [152]. Both the seeds and leaves of *Amaranthus* have valuable nutritional content [65]. The therapeutic and food potentials of the genus (Table 1) make it valuable for conservation priority. In people living with HIV, Amaranth grains increased CD4 count and increased nutrient uptake [153]. Hence, forests with *Amaranthus* species need to be protected to ensure that indigenous communities fully exploit its potentials.

Several species of *Corchorus* are utilised as vegetables, but *Corchorus olitorius* L is the most sought-after [154]. It is a leading leafy vegetable in Côte d’Ivoire, Benin, Nigeria, Cameroon, Sudan, Kenya, Uganda and Zimbabwe [154]. Various extracts of *C. olitorius* exhibit antioxidant, wound-healing, antihyperlipidemic, immunostimulant, antitumor, anti-inflammatory, hepatoprotective, antimicrobial, antidiabetic, analgesic, properties and cardioprotective activities [155]. The methanolic extract of *C. olitorius* exhibited good antioxidant [156] and antidiabetic potential [156,157], with polyphenols being proven responsible for these activities [156].

*Spider plant* (*Cleome gynandra*) is a wild edible vegetable [158,159], highly utilised as a traditional leafy vegetable across Africa and considered a rich, natural food source [160]. It is among the important indigenous African vegetables with enormous potential to contribute to food and nutritional security in sub-Saharan Africa [161]. Hence, it can greatly promote nutrition, good health and well-being if included in the diet, particularly in rural and urban areas of Africa [161].

Some wild forest climbers and trailers are utilised as food and medicine and are culturally valuable tools. Various species of the family Cucurbitaceae fall into this category. Wild Cucurbits are highly utilised across countries in Africa [162,163,164]. Some species are used in the construction of musical instruments and are linked to the history of certain tribes in Africa. Evidence of their ancestral use includes drawings of Cucurbits in some tombs in Egypt. *Citrullus colocynthis* is native to North African countries [165], where it is widespread in the wild. *Citrullus colocynthis* (L.) Schrad. fruit is commonly referred to as bitter melon, bitter cucumber, egusi melon and the bitter vine of Sodom [88]. The seeds are used as food [166]. A wide variety of micronutrients in its fruits and seeds (Table 2) may be beneficial [88]. The consumption of high amount of the plant’s immature fruit is hazardous as it manifests via cerebral congestion, hypothermia, delirium, gastrointestinal irritations, and colitis [167]. 4-methylquinoline, a component of the fruits of *C. colocynthis* is an effective natural insecticide used to prevent weevils in grain storage and the management of spider mites [119].

The genus *Momordica* includes *Momordica charantia* L., *Momordica foetida* Schumach., *Momordica cochinchinensis* (Lour.) Spreng, *Momordica tuberose* (Cogn) Roxb. *Momordica balsamina* L. and *Momordica schinzii* Cogn. *Momordica* species are highly utilised in Africa [168]. The consumption of *Momordica* species as vegetables is primarily concentrated in Africa and Asia, where they hold significant dietary and cultural importance [169]. *Momordica balsamina* is known as African pumpkin, Balsam pear (Eng.); exist in the wild in various southern African countries [170]. The young leaves and fruits are harvested and prepared as vegetables, or cooked with other ingredients and the bitterness of the fruit is appreciated in African traditions which symbolises resilience [170]. *Momordica balsamina* wild populations offer valuable ecosystem services (Table 1) and the plant parts have various bioactive compounds (Table 2).

*Momordica charantia* exists in the wild in various West African countries and has diverse uses. In Togo, *Momordica charantia* is used by many healers who claim that it helps to obtain favours and serves as protection against curses, diseases, evil spirits, spells and madness [171]. According to history, the ancestors of the Togolese Guin lived on the coast of Ghana but later fled intertribal warfare caused by the slave trade and moved to Togo [172]. The Guin ancestors wore a necklace of *Momordica* vines, which repelled their enemies and protected them as they relocated to the northern side of Lake Togo [171]. The young fruits and shoots of *Momordica charantia* serve as supplementary or emergency food in some parts of West Africa [82]. The wild vegetables and fruit trees highlighted in this study are valuable food and medicine resources (Table 1) with valuable nutrient, mineral and bioactive contents (Table 2) and thus play vital roles in forest biodiversity, wild food systems and health.

Wild vegetables and fruits play vital roles in fulfilling the pillars of food security, which are availability, accessibility, utilisation and stability. Forests offer a natural, often readily available, and accessible source of food to those living near the forests. Forest food resources are rich in various micronutrients, vitamins, minerals and bioactive compounds and can improve dietary quality and health (Table 2). Wild fruits and vegetables from African forests improve dietary diversity and help meet essential nutrient needs among vulnerable and disadvantaged communities, such as in rural areas of Africa. Conserving these ecosystems ensures a stable and accessible food source, especially during climate-related disruptions.

According to Clapp et al. [173], to transform the food system, there is a need to expand the scope of food security beyond the four well-documented pillars of availability, access, utilisation and stability to acknowledge sustainability and agency. Agency refers to the ability of individuals and communities to exercise their voice and make crucial decisions about their food systems, which are critical to the functionality of food security and emanating from the ideologies of the right to food legal framework [173]. The right to food is protected under the human rights law, which implies the right of people to feed in dignity [174]. Food justice also considers the global recognition that the food system is linked to public health [175] because of the connection between food, health and the environment [176], making them integral parts of human existence.

According to Fajinmi et al. [177], the functionality of the African traditional medicine system is largely dependent on the availability and sustainable use of African indigenous medicinal plants. Hence, effective biodiversity conservation ensures the sustainable use of medicinal plants in forests and directly supports the functionality of the African traditional medicine system. African forests are a reservoir of valuable medicinal plants used for the treatment of emergency health needs, such as pain, fever, stings, headaches, and stomach aches, among others. Several diseases are treated with plants harvested from the forests, and this practice is an integral part of the African culture. Biodiversity conservation is crucial to preserve the African culture of traditional medicine and indigenous knowledge of the medicinal uses of African wild plants.

**Figure 1:**
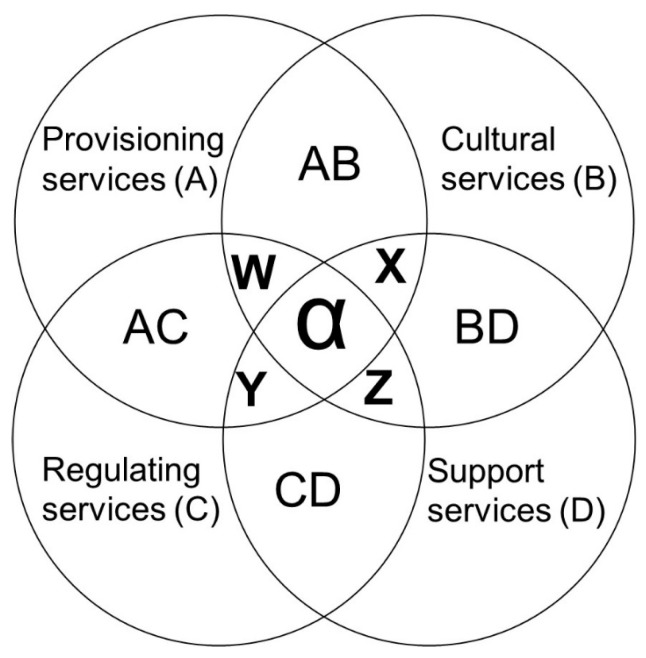
Provisioning services of African forest species.

Provisioning services provide the basic needs for human well-being and survival. It directly supports human survival with tangible goods and resources such as food, water, wood, fibre, pharmaceuticals, and raw materials for industrial products, and it indirectly supports the sustainability of livelihoods. Most wild African leafy vegetables offer provisioning services and regulating services (pollination). Some Cucurbitaceae species used for traditional medicine preparations and food are also used for magical/ spiritual purposes. According to Fajinmi et al. [163], *Momordica charantia* is highly revered in the West African culture for its magical/spiritual relevance. As a result of its ecosystem services (provisioning, cultural and regulating services), it falls in the ‘W’ category of ecosystem services (Figure 1). Wild fruit trees offer more ecosystem services and contribute to the overall health and stability of the forest (Figure 1). For example, Marula trees offer provisioning services (food, fibre and natural medicines), regulating services (climate regulation and pollination), cultural services (cultural diversity, spiritual and religious values), and supporting services (nutrient cycling).

Marula trees offer climate regulation through carbon sequestration and cooling of the environment. Marula trees and some other African wild fruit trees play a significant role in nutrient cycling in the forests. *Sclerocarya birrea* plays this role through various mechanisms such as its deep root system, leaf litter and interaction with other organisms, thus influencing the structure and function of the ecosystem. In addition, Marula is used for cultural practices and spiritual purposes, including chasing away Gremlins (Tokolosi), divination, and banishing evil spirits [178]. Among the Zulus, it is referred to as the marriage tree, and the bark infusion is believed to determine the gender of an unborn child. Considering the numerous uses of the Marula tree, covering the four ecosystem services, it is an alpha (α) forest species. The monkey orange tree, *Dovyalis caffra*, *Parinari curatellifolia*, *Strychnos innocua*, and *S. spinosa*, offer three ecosystem services and fall in category Y (Figure 1). Proper documentation of their cultural/magical uses could help them achieve alpha (α) status. Deforestation could lead to the loss of indigenous knowledge about the food, medicinal, cultural and spiritual values of African indigenous wild plants. A lack of effective conservation effort to protect endangered or vulnerable wild fruit species could change their status from α status to W or Y status (Figure 1). Involving African indigenous communities in the protection of indigenous forests that meet their food, health, cultural and spiritual needs will ensure their right to forest-food, equitable access and use of forests to fulfil other needs, and that biodiversity conservation efforts are inclusive and transparent. Hence, inclusivity and transparency in biodiversity conservation policies could ensure food and social justice in disadvantaged and vulnerable communities surrounding forests.

### 2.4. Contribution of Indigenous People to Conservation Engagements

Africa’s biodiversity is exceptional, occupying 20% of the world’s land and could play a vital role in achieving the UN 30 × 30 target [179]. The African continent has over 1100 species of mammals, 2500 birds, 950 amphibians, about 2000 reptiles, 5000 freshwater fish species and 70,000 plant species [180]. Africa has 8 of the 36 global biodiversity hotspots [181]. Local communities have indigenous knowledge about these species, and some of this knowledge has been passed from one generation to the next. Hence, their involvement in biodiversity conservation could help achieve effective conservation. Biodiversity conservation controlled by external organisations often involves approaches that change local practices and tend to lead to ineffective conservation and negative social outcomes [182]. Since the World Parks Congress held in Durban (2003), the contribution of local people and communities to biodiversity conservation has been recognised [183,184]. Their collaborative effort is perceived to yield more effective biodiversity and conservation outcomes [185,186].

The ecosystems managed by indigenous local people/communities are in better ecological condition and are deteriorating less quickly than those managed by a non-indigenous system [187]. The management of lands by indigenous communities is often inseparable from the culture of the people that manage it [188], incorporating the crucial role of traditional ecological knowledge to create and direct conservation policies and practices [27]. Conservation paradigms have evolved from fortress and exclusionary conservation to community or rights-based conservation strategies [27]. Several biodiversity conservation projects involve collaboration with indigenous people/local communities, yielding successful outcomes.

South Africa’s Biodiversity Stewardship Programme (BSP) envisions active collaborative efforts of private landowners in biodiversity conservation as a tool to achieve the country‘s overall conservation objectives (as formulated in the NBSAP) [189]. This inclusive approach recognises the vital role of private landowners in protecting and maintaining South Africa‘s biodiversity, with various benefits that give landowners/indigenous people a sense of motivation [189]. These benefits include: access to a variety of funding and support structures to assist with conservation efforts; recognition of ecosystem services with significant ecological and economic value enhanced through sustainable land management; opportunities for landowners to collaborate with conservation organisations, and government agencies for knowledge sharing and access to a broader conservation community; and contractual agreements between the government and private landowners with incentives and recognition of the efforts of landowners in conserving biodiversity [189].

In 2003, BSP was implemented, and by 2013, it was established in all nine of South Africa’s provinces. Seventy protected areas had been declared through provincial BSP agreements by 2014, with another 145 under negotiation [190]. Approximately 564,000 ha of protected areas had been declared using BSPs by 2016 [190]. This strategic programme was established to protect the country’s unique and diverse array of species [191]. The BSPs serve as a valuable tool that has offered an opportunity to significantly expand South Africa’s terrestrial protected area coverage to achieve the national and international biodiversity conservation goals under the Convention on Biological Diversity (CBD). Around 68% of protected estates were achieved through the BSP from 2008 to 2016 [192], an indication of the crucial role of communities in achieving effective conservation. According to Dawson et al. [192], equitable conservation involving the empowerment of indigenous peoples and local communities is a primary pathway to efficient long-term biodiversity conservation, especially when endorsed by laws and policies.

Bezeng et al. [179] highlighted two major biodiversity conservation success stories. A partnership between the government, the Conservation Society of Sierra Leone and the United Kingdom (Royal Society for the Protection of Birds) progressed into a 20-year funded project, ‘Reducing Emissions from Deforestation and Forest Degradation in developing countries (REDD+)’. ‘This REDD+ project protects habitats for 327 bird species, 650 endemic plant species, and 49 mammals, including the Pygmy Hippo and the Western Chimpanzee’ [193]. Previously, the Gola Forest Reserve’s status allowed small-scale logging, industrial and artisanal mining, and agricultural activities, exposing the forest to threats and rapid deforestation and degradation [193]. Through the Gola REDD + Project conservation efforts, about 350,000 verified carbon units were generated annually [179].

This project involves efforts from local stakeholders, government, communities and national NGOs to manage this entire landscape sustainably to sustain local communities and wildlife [193]. Carbon finance supports over 160 staffs, the majority of whom inhabit the forest edge communities, through the Gola Rainforest National Park Forest Guard and sustainable cocoa production [193]. Various projects, such as National Park Forest Guard employment opportunities and the establishment of a cocoa farmer’s co-operative, have contributed immensely to the rebuilding of lives after a traumatic decade of civil war and the devastating Ebola outbreak [193]. This partnership has, therefore, substantially contributed to social benefits and biodiversity conservation [179], achieved by fair benefit sharing between the indigenous people and other stakeholders.

Another fascinating conservation report is that of the Shiyanga in Tanzania [179]. The long-term financing benefits to both biodiversity conservation and local communities are evident in Shinyanga, Tanzania [194]. This is by far one of the most unique restorations for conservation efforts with great, impactful outcomes. Before 1985, the region experienced severe degradation of its *Acacia* and Miombo woodlands, partly from cash crop-based agricultural expansion [194]. However, carbon financing projects have facilitated the restoration of more than 300,000 ha of Miombo and Acacia woodlands since 1985 [194]. Overall, this project led to the availability and accessibility of wild food resources and other economically valuable forest resources, equity, and the sustainability of livelihoods. This large-scale reforestation has enhanced the resilience of local ecosystems and strengthened community resilience through sustainable land use practices [194]. A variety of ecosystem services that were lost were restored through the project.

The Kimana Community Wildlife Sanctuary, Kenya’s first community-owned and managed wildlife sanctuary, serves as a flagship for local involvement in tourism [195]. It features diverse habitats, including swamps, savannah plains, and *Acacia tortilis* woodlands [195]. The introduction of the protected area in Kimana has improved local biodiversity and created various opportunities such as social amenities, business opportunities and transportation support [195]. The project has reduced wildlife conflicts and improved safety in the protected area; and authorities support community development programmes like education, health, infrastructure, housing, water provision, and livestock vaccination [195]. The community employs indigenous conservation practices, with 99% of community members acknowledging the role of councils of elders or group ranch committees in managing natural resources, enforcing cultural values, and imposing penalties on offenders [195].

Sghaier et al. [196] highlighted the crucial role of community inclusivity in the Community-based rangeland management in Tataouine (south-east Tunisia). The Chenini rangeland unit is located west of Tataouine Governorate in southern Tunisia and covers 46,606 ha, including both private and collective rangelands. It is bordered by major geographic features like the Great Eastern Erg and the Dhahar Plateau. The area has various medicinal plants which serve as a source of income for the indigenous people around the rangeland. The area is exposed to threats from unsustainable practices despite its high pastoral potential. To address this, technical rangeland management approaches such as resting (temporary grazing exclusion to restore vegetation) and silvo-pastoral planting have been implemented through negotiation with landowners [196]. These efforts are supported by advisory services provided by NGOs, CBOs, and government agents. Assessments of rangeland conditions through field studies and participatory methods reveal that, prior to implementing participatory management, satisfaction with all indicators was low [196]. After adoption, significant improvements were observed across ecological, social and economic indicators; participatory planned grazing reduced grazing pressure, enhanced biodiversity, minimised land degradation and desertification, and improved overall ecosystem services [196]. Social benefits included increased community, youth and women engagement, better interaction with government agencies and improved livelihoods. Economically, the approach boosted rangeland profitability and reduced costs [196]. Researchers suggested that the determination and identification of ecosystem services is a prerequisite to estimating the relative importance of ESs to a community and ensuring their conservation and sustainable management [197]. Sustainable management of the ecosystem to ensure the availability of ESs is crucial for the sustainability of livelihoods and climate change resilience.

### 2.5. Biodiversity Conservation as a Climate Change Resilience Tool

Resilience building is the improvement of system capabilities to predict and absorb disasters and crises and to recover and adapt from such shocks [198]. Such improvement enables households, communities, and countries to shield, restore and improve livelihood systems in the face of threats that impact agriculture, nutrition, food security and food safety [198]. Increasing the resilience of livelihood is an important part of the United Nations (UN) 2030 Agenda for Sustainable Development and its 17 sustainable development goals (SDGs), and the nature-based solutions promoted in 2019 by the UN Climate Action Summit to tackle the increase in climate crises, environmental degradation and food insecurity [188]. Building livelihood resilience and sustainable management of natural resources have emerged as requirements for the humanitarian, development and peace nexus [198]. 

Biodiversity stabilises ecosystem productivity in the long run and is thus considered a crucial tool that supports the resistance and resilience of ecosystem functions to droughts [199,200]. Biodiversity and ecosystem services are part of an overall adaptation strategy to help people adapt to the negative impacts of climate change [201]. The major climate change effects evident in sub-Saharan Africa that negatively impact food security and health are increased temperatures and, consequently, drought. Many regions of the world have investigated the impacts of 1.5, 2 and 3 °C of global warming [202]. Climate change is expected to increase the mean annual and global surface temperature by 1.7 and 3.8 °C, respectively, by 2100 [203]. There is a projected increase in arid conditions, especially in southwestern parts of southern Africa, towards the end of the 21st century [204], attributed largely to an increase in evapotranspiration [205]. 

Furthermore, regional warming is expected to exacerbate and increase the severity of drought in southern Africa before the end of the century [206]. Major water shortages and harsh temperature conditions are being experienced in many parts of South Africa and the SADC region [207], which threaten food supply and agricultural sustainability and limit crop production [67]. The rate at which drought, mean annual and global surface temperature are increasing, presents a frightening future for food supply and agricultural sustainability [67]. Droughts often occur in South Africa, with the more recent drought (2014–2016) harshly reminding people of the need to be more active about droughts [207]. In forest ecosystems, the influence of biodiversity on drought resistance and resilience is attributed to beneficial interactions among tree species, differential stomatal regulation strategies, and selection effects such as competitive dominance of deep-rooted species [208]. Research evidence from subtropical tree communities suggests that the stabilising effect of tree species richness is attributed to interannual variations in the growth of different tree species, which cushions the community against various stress-related growth deteriorations [209]. In periods of severe emergencies or prolonged crises, forests and trees play vital roles in the well-being of vulnerable groups by providing forest foods, fodder and wood fuel as important aids to cope in times of shocks [208]. Several poor and vulnerable communities depend on wild-sourced food during times of drought.

#### 2.5.1. Role of Drought-Tolerant African Wild Leafy Vegetables and Fruits in Sustaining Livelihoods During Drought

Biodiversity alleviates the impact of droughts on rural production with an increase in the natural biodiversity level by one standard deviation compared to the regional mean, significantly reducing the impact of droughts [210]. Biodiversity reduces the negative impacts of drought on rural incomes to almost zero, suggesting that efforts to stop the global biodiversity decline may reduce the vulnerability of rural households to increased weather extremes [210]. Biodiversity conservation can thus play a key role in poverty alleviation in developing countries as the welfare-supporting role of natural resources through ecosystem services is greater in the presence of droughts [210]. Poor people will experience uncertainties about food in a changing climate [28], with non-farming, low-income rural and urban households whose incomes fall below the poverty line facing similar conditions because of climate change impacts [211,212].

Biodiversity provides irreplaceable sources of food, new drugs, and genetic variation, which may have evolutionary importance for pest resistance, pollination and soil fertility [28]. Biodiversity is a natural source of wood for cooking, wild food and fodder, and material for shelter; it conserves water resources and provides other ecosystem services; and it reduces the effects of extreme weather conditions [198]. There is increasing evidence suggesting that maintaining the diversity of foods in forests and trees (such as fruits, nuts, leaves and fodder, mushrooms, seeds, honey, fish and wild meat, and insects) is essential for strengthening food system resilience [198]. Hence, ensuring the diversity of various drought-tolerant plant species used as leafy vegetables, fruits, and fodder for animals in indigenous forests is a drought resilience measure that could help reduce the negative impacts of drought on vulnerable communities.

Amaranths, *Corchorus olitorus* (Jute), *Cleome gynandra*, *Momordica balsamina*, *Momordica charantia* and *Citrullus colocynthis* are widely used wild vegetables in Africa and have been reported by various authors to be drought-tolerant. Amaranth is drought-tolerant [67,213,214,215] and recovers after a long period of severe drought stress [216]. Amaranth grows rapidly under a wide range of unfavourable abiotic conditions, including heat and drought stress, high salinity, acidity, or alkalinity, largely due to its C4 photosynthesis and is thus a promising food crop [66]. *Cleome gynandra* is drought-tolerant [217] and adapts well to diverse habitats (predominantly in warm climates) due to its C4 photosynthetic mechanism [218] which allows the plants to thrive in a wide range of extreme climatic and edaphic conditions [150] including in hot and dry environments [73,219], semiarid, humid and subhumid climates with diverse soil types [73]. Jute mallow is drought-tolerant [103], but most varieties need water for optimum growth. *Momordica charantia* [220,221], *M. basalmina* [220,222] and *Citrullus colocynthis* [223,224,225] are drought tolerant. Similarly, some of the highly utilised, wild African indigenous fruit trees are drought tolerant, a unique potential that makes them crucial tools to mitigate climate change impacts on nutrition and livelihoods.

The Marula tree is widespread in the drier areas where drought is relatively frequent [226], such as bushveld to woodlands [227], indicating its drought-tolerant capacity [227,228]. It forms part of the survival systems of the people during drought [229]. *Dovyalis caffra* tree is resilient, evergreen and productive in adverse conditions such as drought, frost [33,49,230], sea breezes and salt spray [228] and saline environments [49] and thrives in open bushes and wooded grasslands. *Parinari curatellifolia* is highly utilised during challenging periods of household food insecurity [55]. The bark is corky, a characteristic that helps increase protection against high temperatures, drought conditions, and wildfires [123]. *Strychnos innocua* and *Strychnos spinosa* are among the twenty species that produce edible fruit in drought-prone and semi-arid areas out of around seventy-five *Strychnos* species recorded in Africa [35,60]. The trees of *S. spinosa* [231,232] and *S. innocua* [232,233] are typically found in semi-arid and drought-prone environments where trees may still produce fruit even during water shortages [232]. Apart from the role of conserved forests as a source of food, they also serve as a crucial tool for carbon sequestration. Fruit trees contribute significantly to the reduction in atmospheric carbon dioxide through carbon sequestration [234].

#### 2.5.2. African Indigenous Wild Fruit Trees as a Tool for Carbon Sequestration and Climate Resilience

Carbon dioxide emissions and climate change are pressing issues globally. Global warming resulting from various human activities is the greatest concern of the new millennium [235]. Fruit trees significantly reduce atmospheric carbon dioxide through carbon sequestration [234,236] as a result of their structural features [237]. Fruit trees produce oxygen and absorb carbon dioxide, contributing significantly to the oxygen-carbon dioxide cycle [238], converting sequestered atmospheric carbon dioxide into soil and biomass [236]. In the last two decades, 77.36% of African countries had greater forest losses with approximately 32 × 103 kha net loss, resulting in 15.73 Pg C of carbon dioxide emissions, reduced carbon storage and sequestration decreased to −0.69 and −1.37, respectively [236]. Carbon dioxide (CO_2_) and other greenhouse gases released during human activities contribute significantly to climate change [239]. Many studies often overlook the key role of forests in carbon sequestration and climate change mitigation in understanding environmental impacts and sustainability approaches [236].

Forest ecosystems are the greatest carbon sinks, absorbing approximately 2 billion metric tons of CO_2_ annually [240]. African rainforests cover around 13% of Africa’s land mass [241] but store 90% of the continent’s carbon [203], significantly influencing the climate system [241]. Rainforest decline directly affects human well-being [242]. Many African countries and international organisations have recognised the vital role of forests in carbon sequestration and climate change mitigation and are actively involved in forest conservation and restoration initiatives [243]. Trees absorb 0.42 to 0.65 pentagrams of carbon annually [244]. Carbon sequestration is the process of capturing and storing atmospheric carbon dioxide [244] as carbon in biomass, such as tree trunks, branches, foliage, roots, and soils [245]. The major quantities of carbon are reported to be stored in above- and below-ground biomass, dead wood, litter, and soil organic matter [246]. Several factors, such as tree age, leaf area, and photosynthetic competence, have been reported to be the main determinant factors of the carbon-capturing process during photosynthesis [247].

Moreover, the amount of carbon that trees store depends on their age, species, soil type, climate, terrain, and management practices [248]. The rate of carbon storage increases in young trees, but it decreases after reaching the maximum size [249]. Therefore, introducing young trees into forests through animal-related seed dispersal and germination or human efforts through reforestation could help increase the carbon sink ability of indigenous forests. Reforestation efforts involving planting diverse indigenous fruit trees could have a greater carbon sequestration capacity than existing forests. However, preventing existing forests from decline through effective biodiversity conservation and reforestation with younger trees could work synergistically to achieve effective climate change mitigation. Forests contribute to the mitigation of present and future climate change [250]. Africa’s atmospheric CO_2_ levels can be controlled by biodiversity conservation efforts that can sustain Africa’s indigenous forests and reforestation efforts on degraded forests to manage present and future global warming. Conservation of Africa’s native fruit trees is critical not only for ecological health but also for sustaining human well-being in a changing climate. However, to achieve significant outcomes that will reduce climate change impacts through community-based biodiversity conservation projects, the various challenges associated with indigenous people inclusivity must be addressed.

### 2.6. Challenges Associated with Community-Based Biodiversity Conservation Projects

The methods adapted for the conservation and management of biological resources on ancestral territories; rules of access to genetic resources; how benefits from the uses of those resources should be shared are some of the challenges of community-based biodiversity conservation projects [251]. Integrating indigenous views into community-based conservation policies is a major challenge [251]. Community-based natural resource management (CBNRM) projects rely on donor funding, hence, lack of funding is one of the major challenges facing these projects [252]. Corruption and lack of transparency in accounting for funds awarded for projects and distribution of benefits are challenges facing CBNRM [253]. Marginalisation of indigenous communities in the process of natural resources management and the central government retaining the right to make key decisions; and determine the use of resources are other challenges facing CBNRM [254,255]. Challenges of community inclusivity in biodiversity conservation projects include poor governance, threats to livelihoods and negative stakeholder’s relations [256], leadership transitions, rural-urban migration which affects the availability of skilled community members [257].

### 2.7. Conceptual Framework for Biodiversity Conservation in Africa

An effective problem-solving method/policy can only be achieved when it is tailored to the specific needs and circumstances of the people or communities involved. Hence, Africa’s challenges can be solved using African logic and methods by integrating the needs of the African people, African indigenous knowledge and values, history and culture into policies and decision-making processes. Prioritising equitable access to forest resources, food security, respect for cultural values, poverty alleviation, and sustainable livelihoods is essential for effective conservation. These factors directly impact human reliance on natural resources and their willingness to protect them. Thus, conservation efforts that neglect these factors may face challenges in achieving long-term sustainability and could lead to food and social injustices. Limited access to food and increased food prices are social issues that negatively impact marginalised and vulnerable communities, prompting many African rural communities to rely on forest resources for food. The overemphasis on agricultural systems at the expense of conserving nutrient-rich forest foods has worsened the situation. Hence, a conceptual framework for effective biodiversity conservation that will yield significant impacts on the African indigenous population is crucial.

**Figure 2:**
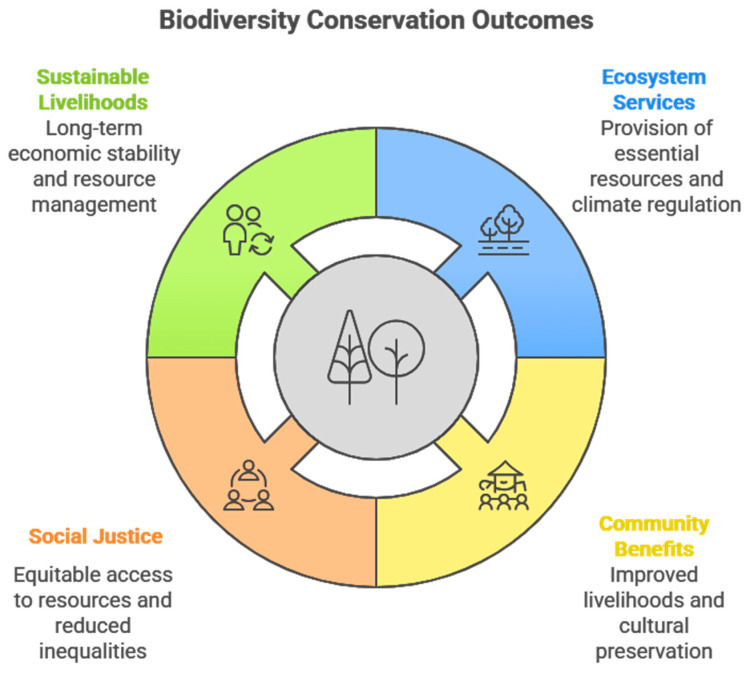
Evident biodiversity conservation outcomes resulting from community inclusion.

Synthesis of literature reflects that community participation in the protection of forests through indigenous knowledge sharing could help achieve effective biodiversity conservation, which in turn offers various ecosystem services such as provisioning services (food and nutritional security, natural medicines, ornamental resources, fodder for animals); regulating services (climate mitigation and resilience); and cultural services (cultural tools, spiritual and religious values). The forest’s ecosystem services are significantly influenced by species richness and diversity (both flora and fauna). Higher biodiversity generally leads to more robust and resilient ecosystems that can provide more valuable essential services. Social justice policies integration into biodiversity conservation policies could ensure equitable access to forest resources, thereby allowing marginalised communities to significantly benefit from ecosystem services (Figure 2). Benefits such as improved livelihoods and cultural preservation among other advantages of community inclusive biodiversity conservation leads to long-term economic stability in previously marginalised, vulnerable households and prompts active resource management; overall enhancing sustainable livelihoods through the synergy of ecosystem services and various financial benefits (Figure 2). The interconnection of social justice, sustainable livelihoods, ecosystem services and community benefits (Figure 2) are crucial for the health and sustainability of forests. High species richness and diversity lead to a more complex and interconnected ecosystem, crucial for climate regulation, nutrient cycling, wild food production and resilience to disturbances.

Forest disturbances could negatively impact forest health and thus disturb the complex interaction between its various forest components. These disturbances can alter forest structures, species composition, ecosystem processes, carbon storage, and biodiversity. For example, the diversity of fruit trees in the forest impacts the existence and functioning of other forest species. Marula trees are dioecious; male trees produce flowers only, while female trees produce fruits, a distinct division of roles that maintains pollination and the provision of food. Hence, the excessive use of male trees for firewood could affect pollination and honey production. Overexploitation of female trees for wood could affect the availability of fruit and oil derived from the seeds. Hence, the occurrence of both male and female trees ensures pollination, which is essential for fruit production and indirectly contributes to honey production by providing nectar and pollen for pollinators. Overexploitation of both male and female plants for firewood can also affect the availability of fruits, oil and leaves for fodder. In addition, Mopane worm which hatches on Marula tree leaves have served as a valuable food resource in some parts of South Africa, especially the Limpopo Province and Marula tree population decline in the wild could affect the availability of this highly nutritious food resource. According to the Kruger National Park, warthogs, elephants, waterbucks, giraffes, and kudu, all eat the fruit and leaves of the Marula tree. Similarly, monkeys, antelope and baboons eat the fruits of *Dovyalis caffra*. Deforestation that affects Marula and Kei apple trees will negatively impact these animals in the wild. The loss of these trees can lead to starvation, habitat loss, increased competition, and a decline in the population of wild animals. Therefore, incorporating plant-animal interactions into biodiversity efforts could help yield positive biodiversity conservation outcomes.

**Figure 3:**
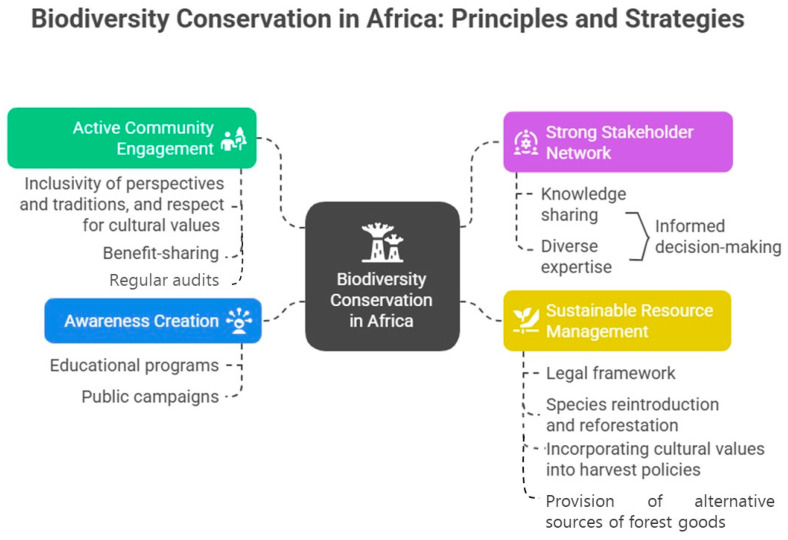
Factors responsible for effective biodiversity conservation.

A conceptual framework for effective biodiversity conservation in Africa should highlight integral principles of forest conservation suitable for the African people. These integral principles are: active community engagement, a strong network of stakeholders, sustainable plant resources management practices, and the creation of awareness through various platforms (Figure 3). Active community engagement can be achieved through inclusivity, which ensures that the perspectives of the indigenous communities are considered and valued. Inclusivity ensures a stronger social bond between indigenous communities and other stakeholders. Indigenous people are more likely to be proactive and invested in conservation projects where their cultural, spiritual, and economic well-being is recognised and supported. When projects acknowledge and respect their values, beliefs and traditional knowledge, indigenous people are more motivated to participate and contribute (Figure 3). This proactive engagement is often driven by a desire to benefit from the project both directly and indirectly through the preservation and promotion of their culture and returns that meet their financial household needs.

These create more interest in protecting the natural resources that support their livelihoods. Many successful biodiversity conservation projects incorporate benefit-sharing (Figure 3) mechanisms such as employment opportunities and fair distribution of proceeds to facilitate community support and engagement in conservation efforts. Proceeds are often used for development projects, infrastructure improvements or other key community needs, further strengthening the link between conservation and community well-being. These strategically empower local communities and increase the sustainability of conservation initiatives. Regular audits of community-based biodiversity conservation projects (Figure 3) such as financial tracking, activity monitoring and conflict-of-interest disclosures can significantly enhance community contributions and prevent corruption. These measures could help to promote transparency and accountability, foster trust and ensure that resources are used effectively for conservation; identify irregularities or potential corruption and allow for timely interventions to prevent further damage. Analysing community members’ perceptions of a biodiversity conservation project is a crucial step to identify potential challenges. Understanding their satisfaction levels and perspectives on the projects’ progression can reveal communication gaps, potential conflicts and other challenges that could hinder its success.

A robust network of stakeholders in biodiversity conservation (Figure 3) significantly boosts knowledge sharing and leads to more informed decision-making, improved conservation strategies and, ultimately, positive biodiversity outcomes. A strong network of stakeholders is crucial for the timely identification of viable sources of income through the monetization of forest goods and services. Africa forests are rich sources of natural resources that can be leveraged for financial gains while promoting sustainable development. A healthy ecosystem could offer ecotourism opportunities and various non-timber products that can be monetized. The development of innovative products from forest resources and ecosystem services, and identifying viable markets, requires a collaborative approach involving a strong network of stakeholders such as researchers, businesses, local communities, and government entities, all working together to identify opportunities, develop solutions, and access local and international markets. Sales and export of non-timber forest products such as organic teas sourced from wild tea plants, organic Aloe gel sourced from wild Aloes, Marula liquor processed from wild Marula fruits, organic Thaumatin from *Thaumatococcus danielli* fruits, organic honey from forest bee hives, organic shea butter from wild shea butter trees, bottled palm wine from wild palm wine trees, miracle berry powder sourced from wild miracle berries, bitter melon teas sourced from leaves of wild *Momordica charantia* or *Momordica balsamina*, and nuts sourced from wild nut trees could form strings of sources of sustainable income that could subsidise government funding and help to achieve sustainable forest management. Portion of revenue generated from sustainably harvested forest products, resources and ecosystem services can be channelled into biodiversity conservation funds to support and sustain conservation projects. This could solve the problem of funding often encountered when government and international agencies funds are no more available to continue biodiversity conservation projects. Hence, this network is a crucial tool for biodiversity conservation projects to progress from depending on external funding to the self-funded stage.

A combination of diverse perspectives and expertise facilitates the exchange of valuable information, experiences and best practices, thus creating a valuable knowledge resource for all members of the group and enabling informed decision-making (Figure 3). Increased knowledge about the medicinal value of wild plant species in a robust stakeholder group could help in the conservation of highly valuable medicinal species and formulation of innovative traditional medicine products that could gain international recognition, supporting the livelihoods of households within the community. For example, the seeds of Amaranth have great anticancer activity, but some African countries consume the leaves only and store the seeds for cultivation. This action is a result of their limited knowledge of the medicinal potential of Amaranths. The increasing incidence of cancer and diabetes can be strategically reduced through the formulation of traditional functional foods such as incorporating Amaranth grains into porridge and consumption of bitter melon tea.

Increased knowledge emanating from a robust stakeholder group can also help to have a deeper understanding of plant-animal interaction and its vital role in achieving successful biodiversity conservation (Figure 3). Plant-animal interactions play an essential role in the sustainability of food systems within African indigenous forests. These interactions include pollination, seed dispersal, and pest control and are vital to maintaining the health and productivity of plant and animal populations, ultimately enhancing food security and nutritional diversity. A robust network of stakeholders in biodiversity conservation, such as scientists (ethnobotanists, ecologists, zoologists and environmental scientists), landowners, local communities, and government representatives can share deeper knowledge about reforestation, carbon sequestration, and other climate-related initiatives that can contribute to efforts to reduce greenhouse gas emissions and mitigate the impacts of climate change.

This strong network can promote sustainable resource management by facilitating the identification of declining populations through fieldwork and community engagement, development of optimum propagation methods, supporting reforestation efforts and advocating for policies promoting sustainable practices (Figure 3). The reintroduction of declining plant species in conservation efforts (Figure 3) can help increase species diversity and help restore ecological balance. A strong legal framework prohibits the indiscriminate use of forest resources and prevents the overexploitation of endangered and declining plant species, thus preventing environmental degradation and social inequality. Cultural values can also be employed to avoid the excessive harvest of forest goods. It is a common principle in many African cultures, especially in rural areas, to leave behind some wild vegetables, fruits and wood during harvest to ensure that resources benefit the whole village/community. This practice allows the environment to replenish itself, promotes community unity and ensures that future generations benefit from its sustainable use. This cultural principle should be well-emphasised in community conservation projects to achieve positive outcomes. Constant et al. [258] highlighted sustainable traditional harvesting methods practised in Vhavenda villages to ensure biodiversity conservation. The various methods of forest plant foraging allow for the survival of the forest species and foster respect and unity among community members. Sustainable management of forests can also be achieved through the provision of alternative sources of forest goods that are often in high demand, such as community farmland and funds for cultivating fast-growing trees for wood production, rather than harvesting from the wild.

Creating awareness about the consequences of deforestation is a crucial step towards preparing the present and future generations to make collective efforts both locally and nationally to conserve indigenous forests. This can be achieved through education curricula, media and social media platforms, organising of festivals and so on. The inclusion of organisations/groups involved in deforestation in the process of their projects, such as mining and construction companies in reforestation engagements is crucial. Research indicates that younger trees generally sequester carbon at a faster rate than older trees, making reforestation efforts incorporating a variety of young trees a potentially effective strategy for mitigating rising atmospheric CO_2_ levels as the young trees are in their active growth phase and can rapidly convert atmospheric carbon into biomass. While matured trees play a vital role in carbon storage, young trees are crucial for the initial, rapid uptake of carbon in newly reforested areas. A combination of protected existing mature forests and active engagement in reforestation with diverse young trees could offer the most effective approach to climate change mitigation through forestry. Africa’s reforestation guidelines for mining companies should prioritise indigenous plant species with various ecosystem services, such as plants with medicinal value, serve as potential food resources, are drought-tolerant, and have potential as carbon sinks. This could offer a healthy, sustainable indigenous forest to the future generation with enhanced climate change resilience.

## 3. Conclusions

Food is a fundamental need of humans, essential for survival, growth and overall health. Amaranths, *Cochorus olitorus*, *Cleome gynandra*, *Momordica charantia*, *M. balsamina*, and *Citrullus colocynthis*; *Sclerocarya birrea*, *Dovyalis caffra*, *Parinari curatellifolia*, *Strychnos madagascariensis*, *Strychnos innocua*, and *Strychnos spinosa* are highly utilised African wild indigenous vegetables and fruit trees and they are critical for sustaining diets during drought and times of food scarcity. The degradation of indigenous forests, disrupting local and indigenous food sources for vulnerable communities, is a social injustice. African indigenous wild fruit trees play crucial roles in carbon sequestration and climate resilience. Biodiversity conservation can help to reduce the impacts of climate change on food availability, enrich dietary options, and support resilient wild food systems. To achieve this, the inclusion of indigenous communities and recognition of their knowledge systems in biodiversity conservation engagement are crucial to environmental stewardship and custodianship. The outcomes of successful biodiversity conservation projects involving indigenous communities often transcends sustainable forest management; they generate various economic benefits and significantly improve the livelihoods of local communities. These projects foster economic development of indigenous communities through income generation, job opportunities, access to markets, and tourism opportunities and ultimately contribute to poverty alleviation. The integrated nature of these transformative benefits reduces conflicts and enhances social equity within previously marginalised communities. The various benefits involved motivates indigenous communities to actively participate in these projects and helps to prevent the loss of valuable medicinal plants, protect declining plant and animal species, and help to control present and future climate change impacts.

To achieve effective biodiversity conservation and mitigate the progression of climate change, educating the general population about the complex relationship between the two factors is paramount. The conservation of African forests with wild indigenous leafy vegetables, crops, and fruit trees is vital for the growth of the African bioeconomy and poverty alleviation and achieving Sustainable Development Goals 1 (end poverty) and 2 (zero hunger), 3 (good health and well-being), and 10 (reduced inequalities). Hence, ‘Biodiversity conservation for human well-being’ should be included in the Sustainable Development Goals to ease the burden of hardships in Africa. This goal should strategically focus on the conservation of existing forests and the reforestation of degraded forests to provide forests to enhance sustainable livelihoods. Including drought-tolerant African indigenous leafy vegetables, fruit trees, wild oil and butter-rich trees, such as palm trees and shea butter trees, could further enhance the capacity of this strategic move to contribute to climate resilience, the sustainability of livelihoods, economic development, and the well-being of African rural communities in the future. African biodiversity conservation and reforestation policies should be grounded in ecological and social justice, integrating equity as a core principle. To ensure sustainable livelihoods, these policies must address social and economic inequalities, prioritise the rights of vulnerable communities, and guarantee that conservation efforts enhance rather than undermine local access to forest resources and ecosystem services.

## Data Availability

Data sharing does not apply to this article as no new data were created or analysed in this study.

## Supplementary Materials

The following supporting information can be downloaded at: https://www.mdpi.com/article/10.3390/plants14172649/s1, Figure S1: Summary of literature screening process, and Table S1: Inclusion and exclusion criteria implemented in the literature screening process.
