# Supplementary material for: Biodiversity Conservation, a Crucial Step Towards Food and Nutritional Security, Food Justice and Climate Change Resilience in Africa
Source: Plants (Basel). 2025 Aug 26;14(17):2649. doi: 10.3390/plants14172649 (PMC12430226; doi:10.3390/plants14172649)
Supplement: Supplementary file 1 [file plants-14-02649-s001.zip › plants-3760034-supplementary.pdf]

SUPPLEMENTARY MATERIAL

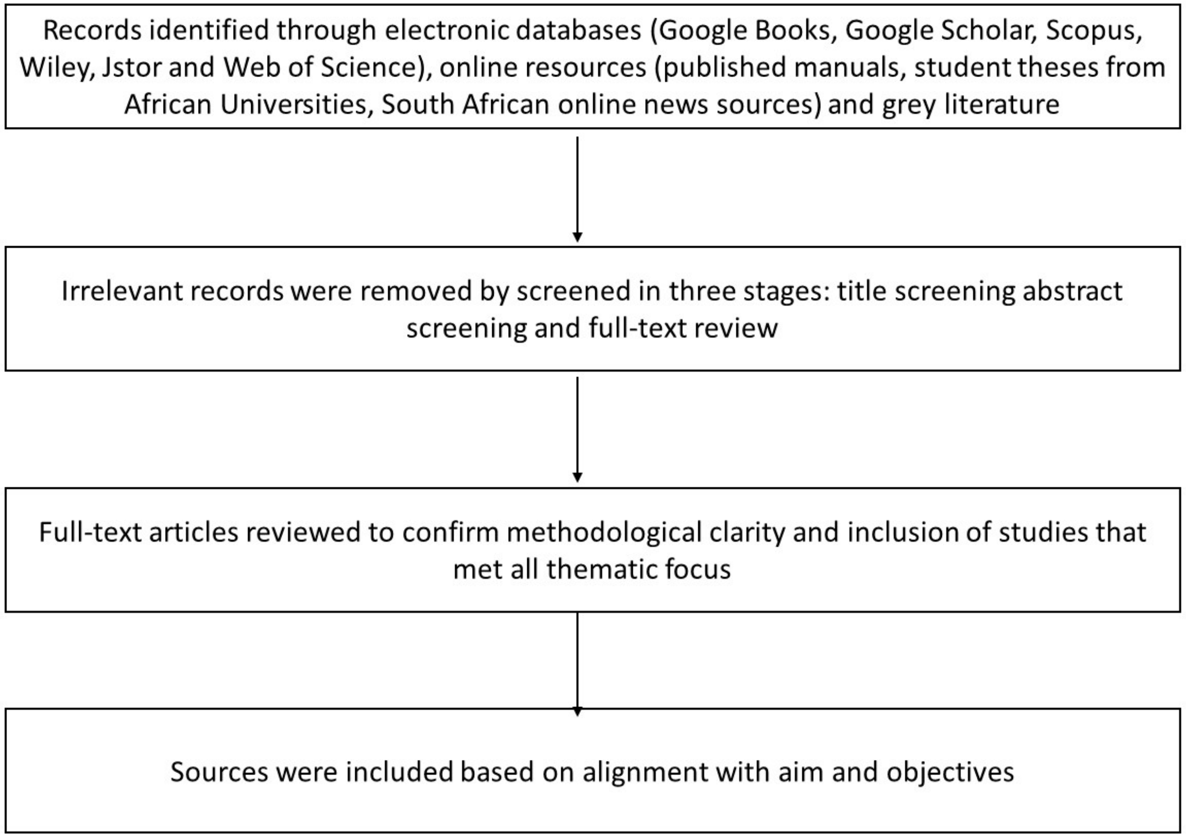

Figure S1: Summary of literature screening process

Table S1: Inclusion and exclusion criteria implemented in the literature screening process

Criteria	Inclusion	Exclusion
Time frame	Records between 2000 and 2025	Published before 2000
Geography	Focus on Sub-Saharan Africa or global studies with sections on Africa except for studies on pharmacological activities	Non-African investigations and reports
Relevance	Direct focus on biodiversity conservation and ecological indicators of success, food and nutritional security, wild African indigenous fruits and vegetables, climate change adaptation and resilience, and studies reporting mineral, nutritional and bioactive contents of highly utilised wild, African indigenous vegetables and their biology activities	Agronomic articles without reference to food and nutritional security, climate resilience and climate adaptation
Type of publications	Peer review articles, institutional reports and grey literature	opinion papers or purely theoretical discussions, studies outside the African context, preprints and duplicates
Methodological transparency	Clearly reported with proper systematic approach and standard empirical and theoretical methods	publications lacking experimental accuracy, research integrity, empirical evidence or measurable outcomes
